# The Configurations of Informal Institutions to Promote Men’s and Women’s Entrepreneurial Activities

**DOI:** 10.3389/fpsyg.2020.01909

**Published:** 2020-07-31

**Authors:** Danish Junaid, Amit Yadav, Farman Afzal, Imran Ahmed Shah, Bharanidharan Shanmugam, Mirjam Jonkman, Sami Azam, Friso De Boer

**Affiliations:** ^1^School of Management and Economics, University of Electronic Science and Technology of China, Chengdu, China; ^2^Department of Information and Software Engineering, Chengdu Neusoft University, Chengdu, China; ^3^Institute of Business and Management, University of Engineering and Technology Lahore, Lahore, Pakistan; ^4^College of Engineering, IT and Environment, Charles Darwin University, Darwin, NT, Australia

**Keywords:** informal institutions, male and female entrepreneurial activities, factor-driven economies, efficiency-driven economies, fuzzy system

## Abstract

While previous studies have examined the impact of informal institutions to determine entrepreneurial activities, this paper explores the different configurational paths of informal institutions to promote men’s and women’s entrepreneurial activities across factor-driven and efficiency-driven economies. We collected data from the Global Entrepreneurship Monitor for 56 countries for the years 2008–2013 and employed fuzzy-set qualitative comparative analysis to conduct the empirical analysis. The results confirm that a single antecedent condition is unable to produce an outcome while combination of different conditions can produce an outcome. We find that cultural-cognitive institutional antecedents in combination with social-normative antecedents create configurations of conditions that lead to the higher levels of men’s and women’s entrepreneurial activities in factor-driven and efficiency-driven economies. Moreover, this study shows that these causal conditions configure differently to promote men’s and women’s entrepreneurial activities in factor-driven and efficiency-driven nations. This paper may create awareness in potential entrepreneurs regarding specific sets of institutional antecedents that can increase the emergence of entrepreneurship in different economic clusters. We show that institutional antecedents which are essential to promote entrepreneurship combine distinctly for men’s and women’s entrepreneurship and this combination varies in different stages of economic development.

## Introduction

Women’s entrepreneurial activities have increased significantly across countries, however the proportion of women’s rate of entrepreneurial activities varies considerably compared to men’s entrepreneurial activities. Kelley et al. (2012) indicate that women entrepreneurs in Pakistan represent only 1% of the total entrepreneurial population while in Zambia women’s engagement in entrepreneurship is 40%. The Global Entrepreneurship Monitor (2017) report states that the number of male entrepreneurs in Portugal represents 10.7% of the adult working age population while only 6.1% are female entrepreneurs. Baughn et al. (2006) argue that informal institutions may promote or restrict women’s participation in entrepreneurial activities. Since some countries associate women’s roles with household activities, while others encourage women to participate in economic activities. The role and status of women are largely contradictory to entrepreneurship in traditional and patriarchal societies of many countries, as societies associate entrepreneurship with males, and depict distinct and contradictory views regarding women’s rights to participate in economic activities (Roomi et al., 2018). However, women’s rate of entrepreneurial activities increases if countries admire and reward them to create entrepreneurial values for society. Thus, institutional heterogeneity demonstrates diverse impacts on men’s and women’s entrepreneurial activities.

Scott (1995) distinguishes informal institutions into cultural-cognitive and social-normative dimensions of institutions. Cultural-cognitive dimension of an institution appears to be a significant predictor of men’s and women’s entrepreneurial activity, as it forms the individuals interpretations and beliefs regarding entrepreneurship (Scott, 1995). Regarding entrepreneurship, it refers to the individuals’ perception of skills, knowledge and experience as well as self-confidence and social capital to create a venture (Valdez and Richardson, 2013). Individuals use cognitive abilities to assemble previously unconnected information that helps them to determine and analyze new products or services, and collect necessary resources to create a new venture (Mitchell et al., 2000). Strong cognitive abilities assist entrepreneurs to undertake feasibility analyses successfully, develop business plans and attract financial capital to establish a new business and grow an existing business (Estrin et al., 2006). While cultural-cognitive institutions reflect the individuals’ cognitive abilities, social-normative institutions are the uncodified values (what is preferable) and norms (how things to be done in line with those values) that are retained by individuals, influencing both the desirability of entrepreneurial activities and entrepreneurship as a career choice (Valdez and Richardson, 2013). In the context of entrepreneurship, social-normative institutions refer to the degree of legitimacy, respect and admiration that are associated with entrepreneurial activities (Baughn et al., 2006). Accordingly, supportive norms are linked with the perception of starting a new business, and determine entrepreneurship as a desirable career choice. Institutional environment that supports and encourages new venture creation generally considers entrepreneurial activities positively and views entrepreneurs as innovators that are essential for economic growth (Danis et al., 2011).

Previous studies have investigated the role of either culture-cognitive or social-normative institutional antecedents in determining men’s and women’s rates of entrepreneurial activities (De Clercq et al., 2010; Koellinger et al., 2013; de la Cruz Sánchez-Escobedo et al., 2014; Gupta and Mirchandani, 2018; Roomi et al., 2018; Santos et al., 2018a). The findings of these studies are inconsistent to promote the rate of men’s and women’s entrepreneurial activities. This might be attributed to the focus of researchers on either set of cultural-cognitive or social-normative institutional antecedents driving men’s and women’s entrepreneurial activities. However, limited attention has been paid to explore the combinatory effects of both cultural-cognitive and social-normative institutional antecedents to promote men’s and women’s entrepreneurial activities. The examination of former and latter institutional antecedents in isolation is unfortunate as it does not considers the integrative and interdependent effects of the institutional context that promote men’s and women’s rate of entrepreneurial activities. This gap in literature is leaving open need to incorporate the configurations of cultural-cognitive and social-normative institutional antecedents to explore the simultaneous interdependencies of the former and latter to start a new business. This may provide a greater understanding how cultural-cognitive and social-normative institutional antecedents combine, complement and substitute each other to promote entrepreneurial activities. It may offer deeper insight how the combinations of different institutional antecedents stimulate men’s and women’s entrepreneurial activities.

Drawing up on Scott (1995) institutional pillars of informal institutions and employing fuzzy-set qualitative comparative analysis (fsQCA) we investigate the combinatory effects of informal institutional antecedents to promote men’s and women’s rate of entrepreneurial activities in different stages of economic development. The empirical counterpart of this study is based upon the data from the Global Entrepreneurship Monitor (GEM) for 56 countries for the years 2008–2013. Economic development can be classified into three stages: (1) the factor-driven stage; (2) the efficiency-driven stage; and (3) the innovation-driven stage (Porter et al., 2002). Factor-driven and Efficiency-driven economies comprise of developing countries while innovation-driven stage includes the most developed countries. The regulatory institutions in developing economies are not congruent with the norms, values and beliefs necessary for entrepreneurship, therefore the emergence of entrepreneurial activities are more likely to occur within the limits of informal institutions (Webb et al., 2014). Thus, *we seek to explore how different combinations of cultural-cognitive and social-normative institutional antecedents promote men’s and women’s rates of entrepreneurial activities in factor-driven and efficiency-driven economies*? *Whether these combinations differ amongst men’s and women’s entrepreneurial activities in factor-driven and efficiency-driven economies?*

This study is structured as follows: Section “Institutional Antecedents and Men’s and Women’s Entrepreneurial Activities” presents the literature review on the effects of informal institutions on entrepreneurship. Section “Materials and Methods” discusses the methodology while section “Results and Analysis” provides the results and analysis. Subsequently, discussion and conclusions are made in section “Discussion.”

## Institutional Antecedents and Men’s and Women’s Entrepreneurial Activities

Institutional environment consists of rules, regulations and social norms and cognitive structures (Scott, 1995) that set the framework to proceed transactions in the market by defining the rules of the game (North, 1990). Institutional environment is considered as structures- starting from rules and regulations to culture, custom and traditions that are operating in a society (Szyliowicz and Galvin, 2010) and largely shape the economic activities. Baumol (1990) argue that entrepreneurial activities are significantly influenced by both formal (rules and regulations) and informal (culture and social norms) institutions. Valdez and Richardson (2013) indicate that informal institutions including cultural-cognitive and social-normative are more likely to promote entrepreneurial activities in comparison to formal institutions. This suggests that cultural values, beliefs and social norms descriptive power in explaining entrepreneurship is higher than rules and regulations. Stephan et al. (2015) argue that cultural values and societal expectations are considered appropriate actions which are based upon dominant and prevalent norms in a given culture or society that foster entrepreneurship. These values and norms establish the ground rules through which members in a society behave (Muralidharan and Pathak, 2017).

Entrepreneurship occurs in a cultural context, thereby appropriate understanding of informal institutions is essential to foster entrepreneurial activities (Williams and McGuire, 2010). Informal institutions determine how societies inculcate values, encourage entrepreneurs and create a cultural milieu that foster entrepreneurship (Puffer et al., 2010). Without proper understanding of informal institutions, institutional reforms introduced by policymakers may have limited impact on overall entrepreneurial activities (Williams and Vorley, 2015). Moreover, informal institutions create individuals’ perceptions, assumptions and judgment of the self, others and their environment that become institutionally embedded and transformed into a social norm of behavior which is difficult to change (Dheer, 2017). Subjective perception of individuals motivates them to identify that an entrepreneurial opportunity exists and can be exploited to gain desirable outcomes, thereby forming the basis of venture creation (Foss et al., 2008). Van Gelderen et al. (2015) argue that entrepreneurial attitudes, motivations and actions are the reflection of the extent to which individuals consider that exploiting an entrepreneurial opportunity and starting a new business are desirable and appropriate. Consequently, institutional apparatus and their effects on entrepreneurial activities widely depend on the cultural framework of the society (De Clercq et al., 2014).

Institutional heterogeneity may explain the different rate of entrepreneurial activities across developed and developing nations. It may provide help in understanding the form and structure of institutional factors that are more or less conducive to the creation of new venture for male and female entrepreneurs. Prior research examines the impact of informal institutions on venture creation (Langowitz and Minniti, 2007), however their influence varies for men’s and women’s entrepreneurs, as both genders socialize differently (DeTienne and Chandler, 2007). Klyver and Grant (2010) indicate that female entrepreneurs are less likely to engage in entrepreneurship compared to male entrepreneurs, as they are less familiar with other entrepreneurs and lack resource providers as well as role models. Women entrepreneurs are more likely to start a necessity-based businesses with less education, limited entrepreneurial skills and experience and are less likely to participate in professional networks than their male counterparts (Hallward-Driemeier, 2013). Moreover, women are less confident about their abilities which further amplifies the adverse impact of their limited skills on entrepreneurship. Consequently, women tend to enter in low-productivity entrepreneurial activities which occur in the informal sector of the economy and concentrate less on high-productivity sectors (Brixiová and Kangoye, 2020). These women are generally less educated and have less capital, and cultural reasons may force them toward necessity-based entrepreneurship which means that fear of failure and good career choice are less important factors in starting a business (Junaid et al., 2019).

Prior studies indicate that the likelihood of pursuing an entrepreneurial career varies between males and females, and that entrepreneurship is widely considered as a male-dominated endeavor (Hughes et al., 2012). A woman’s decision to start a business is influenced by the societal attitudes of an economy (Ahl, 2006). Baughn et al. (2006) indicate that the degree of legitimacy, respect and admiration of women’s engagement in entrepreneurship increase the women’s participation in entrepreneurial activities. Besides normative support, women are also required to negotiate gender roles within society and households to justify their engagement in economic activities (Roomi et al., 2018). The influence of societal attitudes is external, but it exerts substantial impact on the cognitive abilities of an individual (DiMaggio, 1997), as it forms the schemas and beliefs that motivate individuals to perform specific activities and prefer certain activities over others (March and Olsen, 2010). Considering gender-based tendencies regarding entrepreneurship, Croson and Gneezy (2009) argue that, in contrast to their male counterpart, women’s behavior is susceptible to the attributes of the socio-cultural environment. In spite of their importance, our knowledge of how these attributes of socio-cultural environment foster or restrict women’s engagement in economic activities is far from satisfactory (Hughes et al., 2012).

### A Configurational Approach to Promote Entrepreneurial Activity

This section proposes hypotheses which are related to the literature on cultural-cognitive and social-normative institutions and entrepreneurship. Subsequently, a configurational model is put forward to answer the research questions in this study.

#### Cultural-Cognitive Institutions

Cultural-cognitive dimension of an institution reflects the collective understanding of social reality that provides basis for the framing of meaning within a society (Valdez and Richardson, 2013), thereby it develops individuals’ interpretations and beliefs (Scott, 1995; DiMaggio, 1997). Moreover, it shapes individuals attitudes, preferences, motivations and experiences (Yang et al., 2012). Consequently, cultural-cognitive institution not only illustrates significant impact on behaviors of people but also influence the emergence of economic activities in a society (Tsui et al., 2007) including individuals’ engagement in entrepreneurial activities (Liñán and Fayolle, 2015).

Cultural-cognitive dimension of an institution generally reflects the shared social knowledge and individuals’ cognitive abilities that they use to understand entrepreneurship (Kostova and Roth, 2002). These cognitive abilities highlight the individuals’ resources such as the entrepreneurs’ social capital (Shu et al., 2018), prior knowledge and entrepreneurial experience (Frederiks et al., 2019) and fear of failure that may influence entrepreneurial activities. Individuals’ perception of skills and knowledge legitimize the entrepreneurial opportunities for the creation of new venture (Busenitz et al., 2000). Likewise, Saeed et al. (2015) suggest that entrepreneurs’ perception of their ability and confidence toward recognizing an entrepreneurial opportunity increase the occurrence of entrepreneurial activities. Accordingly, entrepreneurs with greater experience and knowledge are more likely to become successful in establishing a new business (Staniewski, 2016). In addition, social networking is also identified as an important determinant of recognition and exploitation of entrepreneurial opportunities (Stenholm et al., 2013). Yousafzai et al. (2015) show how individuals’ social networking and role models as well as their capability to take part in entrepreneurial activity influence entrepreneurship in comparison to regulatory institutions.

Research confirms that women entrepreneurs are also required to develop entrepreneurial skills, knowledge and experience, as well as networking ties to become a successful entrepreneur. Brush et al. (2014) argue that if women develop entrepreneurial skills they can start businesses with greater confidence. Santos et al. (2018b) posits that women may associate in a network of existing entrepreneurs to enhance their level of skills, knowledge and confidence. Since networking provides access to the valuable information, confidence and skills to deal with customers as well as experiences and advice to increase the level of entrepreneurial activities (Santos et al., 2018b). Welsh et al. (2018) indicate that women entrepreneurs tend to be more determined in their entrepreneurial endeavors and risk taking, if they receive support from network ties. Shahriar (2018) postulates that women entrepreneurs are more likely to take risks in a matrilineal society, where women’s propensity to start a business might be higher than men.

Hypothesis 1: The different combinations of cultural-cognitive institutional antecedents promote men’s and women’s entrepreneurial activities.

#### Social-Normative Institutions

Social-normative institutions reflect the collective “sense making” of a society, and demonstrate what is socially favorable and acceptable (Valdez and Richardson, 2013). Normative dimension of an institution deals with the extent of stabilization through the imposition and internalization of societal norms across organizations, individuals and society (Scott, 1995). Following, societal norms individuals aim to act in order to be accepted socially (Scott, 1995). Krueger et al. (2000) argue that social desirability of entrepreneurship as a career choice positively influence the entrepreneurial intentions of potential entrepreneurs, and results in the creation of new venture.

Social-normative institutions reflect values and norms that influence both the social desirability and career options of entrepreneurship. Asante and Affum-Osei (2019) suggest that entrepreneurship as a career option can only be beneficial in the presence of entrepreneurial opportunities, since without business opportunities the existence of entrepreneurship is not possible. Entrepreneurial opportunities arise from the environment in which entrepreneurs operate, and identifying these opportunities may create positive circumstances that lead to the creation of new businesses. Baughn et al. (2006) indicate that in order to seize an existing entrepreneurial opportunity, individuals must be encouraged to engage in entrepreneurial activities. In this respect, media attention tend to have a positive impact on societal norms of a country, as it provides basis to construct the individuals’ understanding that starting a new business is suitable career option (Levie et al., 2010).

The social acceptability of entrepreneurship as a career option for women varies across nations; some societies facilitate or promote women to take part in economic activities while others associate women’s roles with household responsibilities (Achtenhagen and Welter, 2003). Domestic obligations fall disproportionately to women, even if they work longer hours in comparison to their male spouses. Therefore, women may face added complexity to embrace entrepreneurship as a career choice. However, women who are willing to pursue entrepreneurship as a career option may distinguish themselves from others by the set of cognitive abilities. Consequently, potential female entrepreneurs could become more alert to the existing business opportunities. In this regard, previous knowledge is a major factor which influences the perception of business opportunities and exert significant influence on entrepreneurship. Individuals holding knowledge of available opportunities in the market are more likely to exploit these business opportunities in contrast to those who do not have the knowledge. Moreover, Dahal (2013) poses that media stories about successful entrepreneurs positively influence people’s attitudes to business creation, since mass media is recognized as a major factor that reinforces a wide range of attitudes and peoples’ behaviors. Thus, media attention is also expected to exert positive influence on the national rate of women’s entrepreneurial activities.

Hypothesis 2: The different combinations of social-normative institutional antecedents promote men’s and women’s entrepreneurial activities.

**FIGURE 1:**
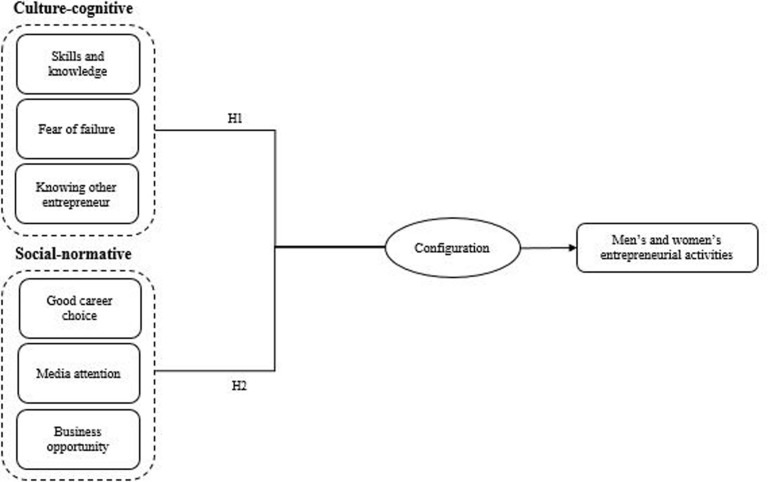
A configurational model.

Based on developed hypotheses, we present the following configuration model in Figure 1.

## Materials and Methods

### Data and Sample

This study considers 6 years of data spanning through 2008–2013 from the GEM Adult population survey. GEM is one of the largest surveys on entrepreneurship and is conducted in more than 100 nations throughout the world. It performs a random national survey of at least 2000 adults of working age population between the age of 18 and 64 years in each country (Reynolds et al., 2005). Data collection is conducted by national academic teams, and the global team supervises the entire process to ensure the quality of the data. Subsequently, GEM harmonizes data to enable cross-country comparison. Our sample considers 56 countries: 25 factor-driven and 31 efficiency driven countries which are listed in Table 1. Porter et al. (2002) classify countries into three stages of economic development. (1) Factor-driven; (2) efficiency-driven; and (3) innovation-driven. The first two stages of economic development include developing nations while third stage comprises of developed countries. Institutional asymmetry influences entrepreneurs to operate outside of formal institutions, but according to the restrictions of informal institutions in developing economies (London et al., 2014). These entrepreneurs are illegal according to the laws and regulations of formal institutions, however they seem legitimate as per prevailing norms, values and beliefs of society’s informal institutions (Webb et al., 2009). Therefore, informal institutions provide greater explanatory power for variations in entrepreneurship in comparison to formal institutions in developing nations. Thus, we decided to explore the association of informal institutions and entrepreneurial activities in factor-driven and efficiency-driven economies.

**TABLE 1:** Sampled countries.

Factor-driven (25)		Efficiency-driven (31)	
Algeria	Philippines	Argentina	Malaysia
Angola	Saudi Arabia	Barbados	Mexico
Bangladesh	Syria	Bosnia and Herzegovina	Panama
Bolivia	Uganda	Brazil	Peru
Egypt	Vanuatu	Chile	Poland
Ethiopia	Venezuela	China	Romania
Ghana	Vietnam	Colombia	Russia
India	Yemen	Costa Rica	South Africa
Iran	Zambia	Croatia	Thailand
Kingdom of Tonga		Ecuador	Trinidad and Tobago
Lebanon		Hungary	Tunisia
Libya		Latvia	Turkey
Malawi		Lithuania	Uruguay
Morocco		Macedonia	Jamaica
Nigeria		Dominican Republic	Guatemala
Pakistan		Serbia	

**Global Entrepreneurship Monitor (2008–2013).**

### Measurement

#### Outcome Variable

In order to capture the impacts of informal institutions on both genders separately, we split our data to distinguish male and female response, but we use a same proxy to measure entrepreneurial activities for both genders. We consider men’s and women’s rates of entrepreneurial activities as the percentage of working age population (between the age of 18 and 64 years) who are in the process of starting a business i.e., a business that is less than 42 months old.

#### Causal Conditions of Informal Institutions

##### Cultural-cognitive

We distinguish male and female responses in order to ascertain the influence of cultural-cognitive dimension separately on men’s and women’s entrepreneurial activities. Cultural-cognitive institutions are constructed using three items from GEM. The first item shows the participants knowledge, experience and skills to start a new business. It can also be viewed as self-confidence in the entrepreneurial domain. It highlights the entrepreneurs’ sense of handling the uncertainty, considering their resources and background within the national context (Valdez and Richardson, 2013). The second item demonstrates the fear of failure that prevents the creation of a new venture. It can be viewed as risk aversion. The third item illustrates whether a potential entrepreneur knows other entrepreneurs before starting a business.

##### Social-normative

As we mentioned earlier, we also discriminate male and female responses while measuring social-normative dimensions of institutions to estimate how these dimensions influence men’s and women’s entrepreneurship. Social-normative institutions are measured by the three items including good career choice, media attention and business opportunities from GEM. The first item shows that most people consider starting a new business is a desirable career option in their country. The second item shows that people often see stories about successful businesses in public media. The third item demonstrates that individuals have good business opportunities in the area where they live preceding the survey in last 6 months. The description of all variables is available in Table 2.

**TABLE 2:** Description of variables.

Variables	Description	Source
Entrepreneurial activities	Percentage of adult age working population who are in the midst of creating a venture or operating an existing business that is less than 3.5 years old	GEM
**Cultural-cognitive**		GEM
Knowing other entrepreneurs	Whether potential entrepreneur knows other entrepreneurs before starting a business in last 2 years	GEM
Skills and knowledge	It shows the entrepreneurs skills, knowledge and experience to start a new business	GEM
Fear of failure	It presents the fear of failure the prevents the creation of new venture	GEM
**Social-normative**		
Good career choice	It demonstrates that individuals consider entrepreneurship is a feasible career choice in their country	GEM
Media attention	People often see stories of successful business in public media	GEM
Business opportunity	Individuals have business opportunities that are worth pursuing in the area where they live	GEM

**GEM, Global Entrepreneurship Monitor.**

### Descriptive Statistics and Correlation Matrixes

Tables 3, 4 present the descriptive statistics and describe the differences between men’s and women’s entrepreneurial activities as well as culture-cognitive and social-normative institutions in factor-driven and efficiency-driven economies respectively. In order to investigate the problem of multicollinearity we calculate variance of inflation factor (VIF) and find that the VIF values of all variables are well below the recommended level of 10 (Kleinbaum et al., 1988; Estrin et al., 2013). Thus, multicollinearity is not a problem in this study.

**TABLE 3:** Descriptive statistics factor-driven economies.

	Mean	SD		Mean	SD
Men’s Entrepreneurial activity	0.224	0.107	Women’s Entrepreneurial activity	0.171	0.122
Knowing other entrepreneur	0.575	0.127	Knowing other entrepreneurs	0.465	0.162
Skill and Knowledge	0.718	0.142	Skill and knowledge	0.623	0.149
Fear of failure	0.322	0.146	Fear of failure	0.353	0.117
Good career choice	0.773	0.102	Good career choice	0.773	0.109
Media attention	0.704	0.149	Media attention	0.715	0.137
Business opportunity	0.593	0.142	Business opportunity	0.542	0.158

**TABLE 4:** Descriptive statistics efficiency-driven economies.

	Mean	SD		Mean	SD
Men’s Entrepreneurial activity	0.152	0.059	Women’s Entrepreneurial activity	0.105	0.663
Knowing other entrepreneur	0.466	0.096	Knowing other entrepreneurs	0.389	0.104
Skill and Knowledge	0.613	0.132	Skill and knowledge	0.507	0.145
Fear of failure	0.338	0.090	Fear of failure	0.401	0.109
Good career choice	0.700	0.110	Good career choice	0.705	0.110
Media attention	0.613	0.129	Media attention	0.630	0.121
Business opportunity	0.441	0.135	Business opportunity	0.405	0.135

Tables 5, 6 represent the correlation matrix of factor-driven economies, we find that women’s entrepreneurial activities have strong positive relationships with knowing other entrepreneurs, skills and knowledge, media attention and business opportunities while women’s entrepreneurship illustrates significant and negative relationship with fear of failure. However, men’s entrepreneurial activities have a strong positive correlation with knowing other entrepreneurs, skills and knowledge and business opportunity and a significant negative relationship with fear of failure.

**TABLE 5:** Correlation among the variables factor-driven economies.

	1	2	3	4	5	6	7
Women’s entrepreneurial activities	1						
Knowing other entrepreneurs	0.637***	1					
Skills and knowledge	0.724***	0.503***	1				
Fear of failure	−0.341**	–0.181	−0.388**	1			
Good career choice	0.097	–0.221	0.369**	0.023	1		
Media attention	0.263*	0.193	0.460***	–0.102	0.526***	1	
Business opportunity	0.584***	0.513***	0.612***	–0.035	0.500***	0.550***	1

*p < 0.05, **p < 0.01, ***p < 0.001.

**TABLE 6:** Correlation among the variables factor-driven economies.

	1	2	3	4	5	6	7
Men’s entrepreneurial activities	1						
Knowing other entrepreneurs	0.529***	1					
Skills and knowledge	0.579***	0.503***	1				
Fear of failure	−0.272*	–0.181	−0.388**	1			
Good career choice	0.036	–0.221	0.369**	0.023	1		
Media attention	0.150	0.193	0.460***	–0.102	0.526***	1	
Business opportunity	0.478***	0.513***	0.612***	–0.035	0.500***	0.550***	1

*p < 0.05, **p < 0.01, ***p < 0.001.

In Tables 7, 8 show correlation matrix of efficiency-driven economies, we find strong positive correlation with knowing other entrepreneurs, skills and knowledge, good career choice, media attention, and business opportunity with both men’s and women’s entrepreneurial activities while fear of failure is negatively associated with men’s and women’s entrepreneurial activities.

**TABLE 7:** Correlation among the variables efficiency-driven economies.

	1	2	3	4	5	6	7
Men’s entrepreneurial activities	1						
Knowing other entrepreneurs	0.229**	1					
Skills and knowledge	0.564***	0.289***	1				
Fear of failure	−0.188*	–0.134	−0.461***	1			
Good career choice	0.448***	0.048	0.482***	−0.315***	1		
Media attention	0.388***	0.314***	0.151	–0.162	0.514***	1	
Business opportunity	0.675***	0.255**	0.616***	−0.462***	0.593***	0.464***	1

*p < 0.05, **p < 0.01, ***p < 0.001.

**TABLE 8:** Correlation among the variables efficiency-driven economies.

	1	2	3	4	5	6	7
Women’s entrepreneurial activities	1						
Knowing other entrepreneurs	0.302***	1					
Skills and knowledge	0.521***	0.289***	1				
Fear of failure	−0.197*	–0.134	−0.461***	1			
Good career choice	0.459***	0.0484	0.482***	−0.315***	1		
Media attention	0.507***	0.314***	0.151	–0.162	0.514***	1	
Business opportunity	0.697***	0.255**	0.616***	−0.462***	0.593***	0.464***	1

*p < 0.05, **p < 0.01, ***p < 0.001.

### FsQCA

We use fsQCA to estimate the combinatory effects of different combinations of conditions on outcome (entrepreneurial activity). In entrepreneurship research, fsQCA is becoming popular (Nikou et al., 2019), as it builds on fuzzy-sets and fuzzy-logic principles with QCA (Ragin, 2000), and its robust analytical approach permits the examination of situations in which the combinations of several different conditions can predict an outcome. Ragin (2013) suggests that fsQCA establishes the association between causal conditions and outcome in terms of sets instead of variables, and its underlying theoretical assumption considers that more than one combinations of different conditions can produce same outcome (Mas-Tur et al., 2015). FsQCA follows the idea of equifinality which suggests that numerous configurational paths can lead to a desired outcome (Fiss, 2007), and also allows that different combinations or sets of causal conditions predict the same outcome. Equifinality and asymmetric causality are two key factors that reveal the complex causal structures of small, medium and even larger samples to conduct the analysis (Silva and Goncalves, 2016).

QCA presents idea that conditions are the clusters of interconnected conditions (variables) which should be simultaneously understood as a holistic integrated pattern, offering dual benefits (Fiss, 2011). Firstly, it assumes asymmetric relationship between independent and dependent variables, such as a variable can be considered necessary but not sufficient for the occurrence of an outcome. Secondly, it measures the impact of a condition on the outcome, in case the presence or absence of another condition is considered to be important (Woodside, 2013). Consequently, conditions combine differently in order to predict an outcome (Mas-Tur et al., 2015). FsQCA presents results in the form of one or multiple configurations which reflect combinations of different causal conditions that produce an outcome. Unlike regression, fsQCA allows researchers to include/exclude a condition from analysis, and explains how multiple combinations of causal conditions collectively contribute to the outcome (Fiss, 2011). We consider fsQCA is valuable for this study as it explores how cultural-cognitive and social-normative institutional antecedents collectively promote men’ and women’s entrepreneurial activities.

### Calibration

We transform the continuous values of datasets into fuzzy set-membership scores by calibration to produce values ranging from 0 to 1 (Ragin, 2009). Following Lewellyn and Muller-Kahle (2016) we calibrate our conditions into three different threshold levels: full non-membership, crossover point and full membership. We consider the 85th and 15th percentile of original data as fully in and fully out respectively, while median is used as a crossover point. Tables 9–12 present the calibration of the cultural-cognitive and social-normative institutional antecedents in both factor-driven and efficiency-driven economies.

**TABLE 9:** Calibration of all variables for the factor-driven economies for male entrepreneurial activities.

Membership	Fuzzy-set value	Knowing other entrepreneurs	Skills and knowledge	Fear of failure	Good career choice	Media attention	Business opportunity	Entrepreneurial activities
Fully in	85th percentile	0.725	0.845	0.391	0.861	0.819	0.740	0.324
Cross-over	Median	0.556	0.722	0.301	0.793	0.751	0.557	0.181
Fully out	15th percentile	0.461	0.614	0.204	0.670	0.572	0.481	0.142

**TABLE 10:** Calibration of all variables for the factor-driven economies for female entrepreneurial activities.

Membership	Fuzzy-set value	Knowing other entrepreneurs	Skills and knowledge	Fear of failure	Good career choice	Media attention	Business opportunity	Entrepreneurial activities
Fully in	85th percentile	0.655	0.778	0.440	0.868	0.826	0.743	0.324
Cross-over	Median	0.443	0.652	0.360	0.796	0.733	0.550	0.156
Fully out	15th percentile	0.337	0.459	0.233	0.636	0.582	0.398	0.044

**TABLE 11:** Calibration of all variables for the efficiency-driven economies for male entrepreneurial activities.

Membership	Fuzzy-set value	Knowing other entrepreneurs	Skills and knowledge	Fear of failure	Good career choice	Media attention	Business opportunity	Entrepreneurial activities
Fully in	85th percentile	0.576	0.740	0.413	0.817	0.758	0.564	0.208
Cross-over	Median	0.453	0.627	0.338	0.697	0.620	0.437	0.155
Fully out	15th percentile	0.375	0.458	0.264	0.599	0.496	0.271	0.087

**TABLE 12:** Calibration of all variables for the efficiency-driven economies for female entrepreneurial activities.

Membership	Fuzzy-set value	Knowing other entrepreneurs	Skills and knowledge	Fear of failure	Good career choice	Media attention	Business opportunity	Entrepreneurial activities
Fully in	85th percentile	0.513	0.633	0.512	0.809	0.761	0.525	0.157
Cross-over	Median	0.365	0.518	0.386	0.695	0.626	0.411	0.092
Fully out	15th percentile	0.289	0.357	0.306	0.612	0.517	0.247	0.044

Subsequently, we compressed the data into the “Truth table” to obtain all expected configurations of antecedents and causal conditions that may promote entrepreneurial activities. The truth table identifies the antecedent or causal conditions that are necessary or sufficient to produce an outcome. Accordingly, fsQCA configurational models illustrate the different combinations of antecedents or causal conditions that are likely to promote the entrepreneurial activities.

## Results and Analysis

The confirmation of each hypothesis is based upon the consistency and coverage values of each configurational model that falls within the recommended range. A configurational model is only informative if its consistency and coverage values are above 0.74 and less than 0.65, respectively, which show the existence of both subset relations and sufficient conditions (Tuo et al., 2019). In addition, raw coverage values determine the empirical significance of a solution and estimate the degree of each configuration model explaining the outcome. Moreover, the unique coverage determines the proportion of a membership in the outcome or the fraction of cases that are highlighted by a single configuration. A high coverage value demonstrates that a configurational model explains the greater amount of the entrepreneurial activities (Fiss, 2011). After considering the consistency and coverage values of each variable, it might be considered as an antecedent or a causal condition of any of the configurational model that is likely to predict an outcome.

**FIGURE 2:**
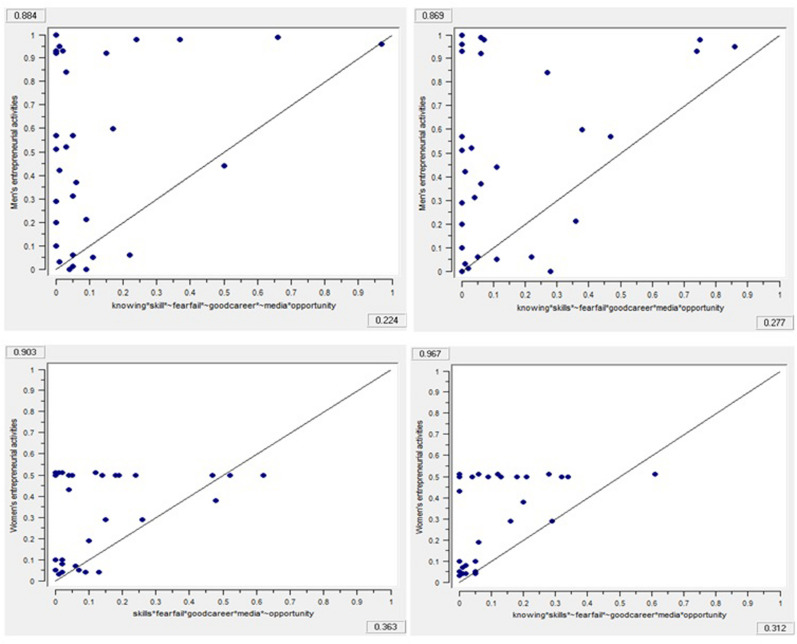
Fuzzy outcome scatterplots associated with results in Table 15.

**FIGURE 3:**
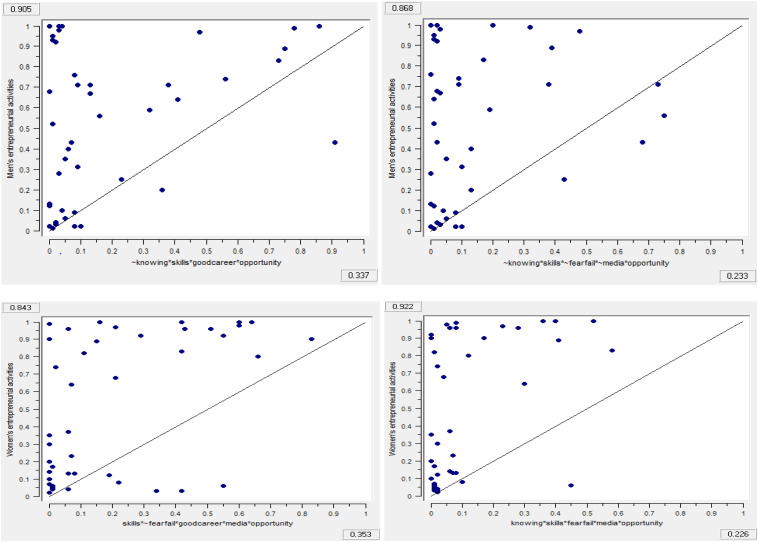
Fuzzy outcome scatterplots associated with results in Table 16.

**FIGURE 4:**
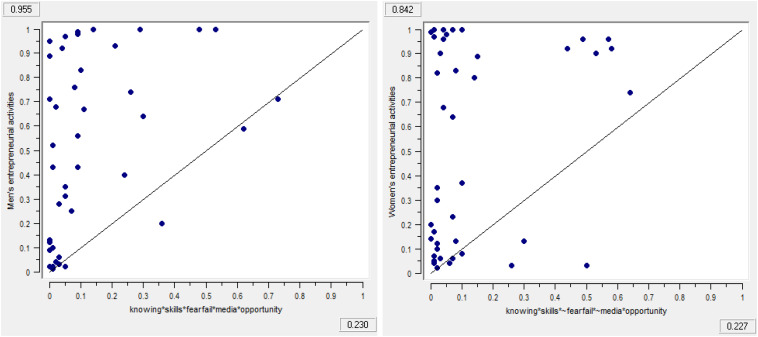
Fuzzy outcome scatterplots associated with results in Table 16.

FsQCA allows exploration of whether the given condition is necessary or sufficient to promote men’s and women’s entrepreneurial activities. Tables 13, 14 present the results for necessity and sufficient conditions for factor-driven and efficiency-driven economies respectively. Necessity conditions are always present whenever an outcome occurs, however whenever the sufficient conditions occur the outcome will be generated. We consider that a causal condition or combinations of different conditions are necessary or sufficient if they demonstrate consistency and coverage values greater than 0.90 and 0.85, respectively (Ragin, 2006). Results in Tables 13, 14 show that neither the presence nor the absence of any causal condition is individually necessary or sufficient to promote men’s and women’s entrepreneurial activities. The presence and absence of each individual causal condition is well below the threshold level to consider it as necessary or sufficient to promote men’s and women’s entrepreneurial activities. These findings support our framework suggesting that cultural-cognitive and social-normative institutions work in combination and substitute and complement each other in promoting men’s and women’s entrepreneurial activities. Thus, to explain and understand how the cultural-cognitive and social-normative institutions jointly promote men’s and women’s entrepreneurial activities, we employ fsQCA to identify the sufficient configurations. We present Figures 2–4 to summarize the findings of the sufficiency analysis for each causal configuration path to promote men’s and women’s entrepreneurial activities in factor-driven and efficiency-driven economies respectively.

**TABLE 13:** Necessity and sufficiency tests in factor-driven economies.

Conditions	Factor-driven male		Factor-driven female	
	consistency	coverage	consistency	coverage
**Cultural-cognitive**				
Knowing other entrepreneur	0.655	0.666	0.828	0.580
∼Knowing other entrepreneur	0.539	0.546	0.612	0.382
Skills and knowledge	0.702	0.681	0.880	0.589
∼Skills and knowledge	0.438	0.466	0.544	0.358
Fear of failure	0.509	0.509	0.663	0.430
∼Fear of failure	0.646	0.664	0.720	0.484
**Social-normative**				
Good career choice	0.605	0.589	0.734	0.462
∼Good career choice	0.602	0.637	0.617	0.427
Media attention	0.601	0.595	0.769	0.474
∼Media attention	0.523	0.544	0.543	0.385
Business opportunity	0.743	0.676	0.801	0.540
∼Business opportunity	0.424	0.486	0.624	0.403

**∼ Indicates the negation of the condition.**

**TABLE 14:** Necessity and sufficiency tests in efficiency-driven economies.

Conditions	Efficiency-driven male		Efficiency-driven female	
	Consistency	Coverage	Consistency	Coverage
**Cultural-cognitive**				
Knowing other entrepreneur	0.613	0.596	0.606	0.640
∼Knowing other entrepreneur	0.573	0.503	0.537	0.512
Skills and knowledge	0.752	0.703	0.734	0.745
∼Skills and knowledge	0.465	0.423	0.429	0.424
Fear of failure	0.534	0.489	0.488	0.663
∼Fear of failure	0.666	0.619	0.656	0.663
**Social-normative**				
Good career choice	0.731	0.682	0.734	0.745
∼Good career choice	0.477	0.434	0.435	0.431
Media attention	0.697	0.675	0.698	0.734
∼Media attention	0.497	0.437	0.461	0.441
Business opportunity	0.829	0.729	0.834	0.796
∼Business opportunity	0.389	0.378	0.335	0.353

**∼ Indicates the negation of the condition.**

In Tables 15, 16, all the configuration models show that consistency values are more than 0.74, and coverage values are less than 0.65. Thus, the antecedents that are creating configurations are sufficient to promote men’s and women’s entrepreneurial activities (Ragin, 2009). Table 15 demonstrates that the findings of factor-driven economies where model 1M is the combination of knowing other entrepreneurs, skills and knowledge and business opportunity with the lack of fear of failure, good career choice and media attention. On the other hand, model 1F is the combination of knowing other entrepreneurs, skills and knowledge, good career choice and media attention with the absence of business opportunity. We notice that absence of fear of failure, good career choice and media attention with the presence of business opportunity in model 1M is functionally equivalent to the presence of fear of failure, good career choice and media attention along with the absence of business opportunity in model 1F.

**TABLE 15:** Configurations for entrepreneurial activities in factor-driven economies.

Conditions	Factor-driven			
	Male		Female	
	1	2	1	2
**Cultural-cognitive**				
knowing other entrepreneur	•	•	•	•
Skills and knowledge	•	•		•
Fear of failure	⊗	⊗	•	⊗
**Social-normative**				
Good career choice	⊗	•	•	⊗
Media attention	⊗	•	•	•
Business opportunity	•	•	⊗	•
**Consistency**				
Raw coverage	0.223	0.277	0.362	0.311
Unique coverage	0.145	0.199	0.198	0.154
Overall solution consistency	0.884	0.868	0.902	0.966
Overall solution coverage:	0.472		0.562	

*•*Indicates presence, ⊗ indicates absence.**

**TABLE 16:** Configurations for entrepreneurial activities in efficiency-driven economies.

	Efficiency-driven					
Conditions	Male			Female		
	3	4	5	3	4	5
**Cultural-cognitive**						
knowing other entrepreneur	⊗	⊗	•		•	•
Skills and knowledge	•	•	•	•	•	•
Fear of failure		⊗	•	⊗	•	⊗
**Social-normative**						
Good career choice	•			•		
Media attention		⊗	•	•	•	⊗
Business opportunity	•	•	•	•	•	•
**Consistency**						
Raw coverage	0.337	0.232	0.229	0.352	0.225	0.226
Unique coverage	0.088	0.044	0.071	0.093	0.020	0.006
Overall solution consistency	0.904	0.868	0.954	0.842	0.922	0.841
Overall solution coverage:	0.664			0.635		

*•*Indicates presence, ⊗ indicates absence.**

Model 2M is the combination of knowing other entrepreneurs, skills and knowledge, good career choice, media attention, and business opportunity and lacks fear of failure. Model 2F is a combination of knowing other entrepreneurs, skills and knowledge, media attention and business opportunity with the absence of fear of failure and a good career choice. Remarkably, the presence of good career choice in model 2M is functionally equivalent to the absence of good career choice in model 2F.

Table 16 shows the findings of efficiency-driven economies. Model 3M suggests that skills and knowledge, good career choice, and business opportunity in combination with the absence of knowing other entrepreneurs can predict men’s entrepreneurship whereas model 3F requires the combination of skills and knowledge, good career choice, media attention and business opportunity without the fear of failure.

Model 4M requires the presence of skills and knowledge and business opportunity with the lack of knowing other entrepreneurs, fear of failure and media attention. On the other hand model 4F requires the combination of knowing other entrepreneurs, skills and knowledge, fear of failure, along with media attention, and business opportunity. We find that lack of knowing other entrepreneurs, fear of failure, and media attention in model 4M are functionally substitute to the presence of knowing other entrepreneur, fear of failure and media attention in model 4F.

Model 5M requires the presence of knowing other entrepreneurs, skills and knowledge, and fear of failure along with media attention and business opportunity. Model 5F requires the presence of knowing other entrepreneurs, skills and knowledge and business opportunity with the lack of fear of failure and media attention. Model’s 5M presence of fear of failure and media attention act as functionally equivalent to the absence of fear of failure and media attention in model 5F.

Our findings confirm the prepositions 1 and 2 asserting that cultural-cognitive and social-normative institutions may serve as an antecedent condition and jointly promote men’s and women’s entrepreneurial activities. The results of 1–2M and 1–2F in factor-driven economies while 3–4M and 3F and 5F in efficiency-driven economies show the different causal conditions that can promote men’s and women’s entrepreneurial activities. However, models 5M and 4F in efficiency-driven economies illustrate that the same conditions can also promote men’s and women’s entrepreneurial activities.

## Discussion

In this study, we explore how different combinations of both cultural-cognitive and social-normative institutions promote men’s and women’s entrepreneurial activities. The empirical part of this research is based upon GEM for the years of 2008–2013, and employed fsQCA to conduct the empirical analysis. The results reveal that different combinations of cultural-cognitive and social-normative institutions promote men’s and women’s entrepreneurial activities, and these combinations differ largely amongst male and female entrepreneurs in factor-driven and efficiency-driven economies.

### Configurations of Informal Institutions in Factor-Driven Economies

Table 15 presents the results of factor-driven nations where model 1M indicates that entrepreneurs are required to develop networking as it provides novel and essential information that are likely to facilitate both the firm’s risk taking behavior and problem solving as well as decision making in starting a venture (Lioukas and Voudouris, 2020). Moreover, our results suggest that it is essential for aspiring entrepreneurs to develop networking along with skills and knowledge that are substantially helpful in evaluating further business opportunities to create a venture. Experienced entrepreneurs of a business network may hold unique knowledge that may transform essential information to the nascent entrepreneurs to start a business. Makhbul and Hasun (2011) argue that being knowledgeable may support entrepreneurs to become innovative, and triggers new ideas to seize potential entrepreneurial opportunities for venture creation. Staniewski (2016) considers that individuals with a greater level of networking and entrepreneurial knowledge and skills are more likely to succeed in their entrepreneurial pursuits.

Model 2M suggests that the entrepreneurs’ network improves the individuals’ ability to acquire knowledge to identify and exploit new business opportunities for entrepreneurship (Song et al., 2017; Santoro et al., 2018). In addition, our findings highlight that positive societal attitudes which support entrepreneurship as a career option and present a progressive image of entrepreneurship are also required to increase the likelihood of entrepreneurial activities. In this context, the media showing successful entrepreneurs is necessary in order to motivate aspiring entrepreneurs to engage in entrepreneurship. Since potential entrepreneurs are likely to imitate the behavior of successful entrepreneurs that may reinforce entrepreneurship as a career option (Zellweger et al., 2011).

The women’s entrepreneurship model 1F reveals that networking is an essential component for women entrepreneurs since it binds them in a group, and leads them to raise their voices in order to achieve their joint objectives of creating a new venture (Santos et al., 2018b). In this way they can identify and exploit business opportunities which are created by segmented communication and fit well according to the feminine taste (Santos et al., 2018b). We also find that in factor-driven economies many cultural reasons may force women to engage in necessity-based entrepreneurship. These women are less educated, lack formal financing as well as fear of failure and career choices are not relevant factors for them to pursue entrepreneurship (Junaid et al., 2019). In this respect, media representation of female entrepreneurs may positively influence the success of women entrepreneurs (Ruth Eikhof, 2013).

Model 2F suggests that women entrepreneurs should be encouraged to extend their level of networking as it may significantly help them to share knowledge, experience and contacts, which promote innovation and creativity as well as lead to the emergence of entrepreneurship (Santos et al., 2018a). In addition, network members may teach different skills to aspiring entrepreneurs to cope with a difficult situation (Soetanto, 2017), and provide resources and opportunities that would otherwise be unachievable (Gupta et al., 2014). We further augment the findings of Jung et al. (2018) that media role is essential to highlight the critical role of women’s entrepreneurship to transform the socio-cultural environment and enhance the process of marketization in an economy. The former shapes the societal norms to accept entrepreneurship as a career option for females and latter facilitates the transformation of socialist economy into capitalist that may increase the emergence of entrepreneurial activities.

### Configurations of Informal Institutions in Efficiency-Driven Economies

Table 16 presents the findings of efficiency-driven economies where model 3M indicates that entrepreneurs are required to acquire necessary entrepreneurial skills and knowledge to develop new business models which may create new business opportunities for venture creation (Sousa and Rocha, 2019). Hence, the ability of individuals to identify new business opportunity is the main factor of choosing entrepreneurship as a career choice (Asante and Affum-Osei, 2019). Without the existence of business opportunity, the pursuance of entrepreneurship is not possible (Shane, 2000).

Model 4M shows that skills and knowledge in combination with business opportunity are likely to facilitate entrepreneurship. Entrepreneurial skills are likely to mediate the relationship between opportunity recognition and entrepreneurial orientation (Santos et al., 2018a). In this respect, entrepreneurs’ prior knowledge and skills are likely to impact the extent of an opportunity identification, as they influence the entrepreneurs’ feelings and judgment in making decision to start a business (Shane, 2000). Thus, we realize that entrepreneurs’ skills, knowledge and experience may facilitate the identification of an entrepreneurial opportunity to start a venture.

Model 5M shows that individuals who know existing entrepreneurs are more likely to start a new venture since networking reduces the transaction cost, enhances mobility, lowers social exclusion and makes it easier for potential entrepreneurs to access new opportunities as well as resources. In this context Dimov (2010) and Shu et al. (2018) argue that entrepreneurs’ previous knowledge and skills as well as network ties facilitate the recognition of an entrepreneurial opportunity. Moreover, this model reveals if entrepreneurs’ levels of aspirations are high enough or they consider that entrepreneurship may provide greater earning opportunities in comparison to the foregone employment, then fear of failure attracts more investment in the venture creation (Morgan and Sisak, 2016). Meanwhile, our result reveals that mass media coverage is required to influence the wide range of attitudes and behaviors of peoples to change the individuals’ thoughts, values and sentiments that entrepreneurship is worth pursuing (Hindle and Klyver, 2007).

Model 3F indicates that starting a business is socially acceptable for women if they hold the necessary skills, knowledge and experience to engage in entrepreneurial activities (Junaid et al., 2019). Existing literature on women’s entrepreneurship reports that normative support of a country’s institutional environment is the most critical determinant of the emergence of women’s entrepreneurial activities (Baughn et al., 2006; Yousafzai et al., 2015). We provide evidence that the combination of latter factor along with former is a better predictor for the occurrence of women’s entrepreneurship. These results provide an indication that women’s entrepreneurial skills and knowledge are likely to facilitate the normative support for women’s entrepreneurship.

Model 4F shows women entrepreneurs are required to join a network of existing entrepreneurs which may guide and help them to discover new business opportunities as well as build their confidence to ensure that starting a business is a feasible career option. In this context, existing network members are required to enhance the sense of participation and develop the feelings of belongingness with new members which may strengthen the associational ties among network members that would be a source of satisfaction and confidence for new entrants (Sánchez-Franco et al., 2012). Accordingly, these networking ties may help women entrepreneurs to find new market niches which might be untapped by the traditional men-owned enterprises. In addition, female entrepreneur’s representation in a women magazine may also positively influence the women’s perception of entrepreneurship as a feasible and attainable career option, and shape the direction of their entrepreneurial aspirations (Ruth Eikhof, 2013).

Model 5F demonstrates that women entrepreneurs can create a venture by joining a network of existing entrepreneurs since networking compensates the lack of resources and provide advice and social support to exploit business opportunities as well as predicts the success of venture creation (Burt, 2019). We also find that previous knowledge and experience are critical to perceive the attractiveness of an existing business opportunity, as learning and reflecting upon past entrepreneurial experiences grant confidence to the entrepreneurs whether to exploit or ignore an available opportunity. Experienced entrepreneurs possess knowledge to evaluate the attractiveness and profitability of a business opportunity for venture creation.

## Conclusion

This study investigates the role of cultural-cognitive and social-normative institutional antecedents to promote men’s and women’s entrepreneurial activities in factor-driven and efficiency-driven economies. This research introduces a fuzzy-set approach to entrepreneurship that permits comparing configurations of institutional conditions under which men’s and women’s entrepreneurship proliferates. The findings indicate that the configurations of both cultural-cognitive and social-normative institutional antecedents are required to promote men’s and women’s entrepreneurial activities in factor-driven and efficiency-driven economies, and that these configurations differ among men’s and women’s entrepreneurial activities in different economic settings of factor-driven and efficiency-driven countries.

### Implications

#### Theoretical Implications

This study makes following contributions; (1) while previous studies posit that cultural-cognitive and social-normative institutions determine men’s and women’s entrepreneurship (Baughn et al., 2006; Valdez and Richardson, 2013), this study makes a contribution by showing that these institutions are neither necessary nor sufficient in isolation to facilitate entrepreneurship; (2) this study indicates that cultural-cognitive and social-normative combine differently to promote entrepreneurship in different stages of economic development, and this combination varies for men’s and women’s entrepreneurship; (3) in contrast to previous studies which show that the impact of informal institutions differs in developing and developed countries (Danis et al., 2011), we show that the influence of informal institutions also varies in developing nations like factor-driven and efficiency-driven economies; (4) By employing fsQCA to gender based entrepreneurship research, we reveal the joint effects of institutional antecedent conditions, and extend our understanding of how distinct causal conditions combine to explore the reinforcing and substitutive patterns of relationships that promote entrepreneurship.

#### Practical Implications

This study has some practical implications to promote men’s and women’s entrepreneurial activities. The results of this study provides new insights to understand the complexity of values, beliefs and societal norms which are useful to start a venture for potential men’s and women’s entrepreneurs in different economic zones. By employing fsQCA we find that there are different configurational paths to promote men’s and women’s entrepreneurial activities, and we may have to accept that one size fits all policy would not work for both genders and for different economic clusters. Thereby, policies should be designed with respect to the prevailing economic conditions which conform to the existing cultural values and societal attitudes of a country. Further, the configurational approach may provide benefits to potential entrepreneurs in terms of providing them with useful ideas for starting a business successfully. The results of this research provide awareness to the potential entrepreneurs regarding the sets of institutional antecedents that are essential to start a venture for men’s and women’s entrepreneurial activities in factor-driven and efficiency-driven nations. Thus, our configurational approach may assist aspiring entrepreneurs to observe whether an optimal set of institutional antecedents are available that may lead them to engage in economic activities successfully. Finally, this study demonstrates that by employing a configurational approach we can understand the complexities associated with individuals’ cognitive factors and societal attitudes in promoting men’s and women’s entrepreneurship in factor-driven and efficiency-driven economies.

#### Limitations and Future Research

This research provides deeper insight how different configurations of both cultural-cognitive and social-normative institutional antecedents promote men’s and women’s entrepreneurial activities. In this context, future research can compare how the presence and absence of both cultural-cognitive and social-normative institutional antecedents can restrict or promote entrepreneurial activities. Studies can also complement the institutional antecedents of regulatory institutions to examine their influence on entrepreneurial activities. This study shows the combinations of different institutions that are required to promote entrepreneurship in factor-driven and efficiency-driven economies. Future research can also add innovation-driven economies into the analysis, and examine how combinations of different institutional antecedents vary in developing and developed countries. This research used GEM data to measure the antecedents of informal institutions, future research may consider Hofstede’s cultural dimensions to extend the existing study. The generalizability of the results of this study is restricted to developing economies including factor-driven and efficiency-driven as data is generated from GEM. Future research can consider World Bank or World Value Survey (WVS) to add more countries to examine the same phenomena.

## Data Availability Statement

Publicly available datasets were analyzed in this study. This data can be found here: https://www.gemconsortium.org/data.

## Ethics Statement

Ethical review and approval was not required for the study on human participants in accordance with the local legislation and institutional requirements. Written informed consent from the participants was not required to participate in this study in accordance with the national legislation and the institutional requirements.

## Author Contributions

DJ was the main author of this study. AY was remained helpful in drafting the manuscript. FA contributed in the methodology. IS provided valuable help in final write up. Rest of the authors contributed equally to prepare the final version of the article and approved the submitted version.

## Conflict of Interest

The authors declare that the research was conducted in the absence of any commercial or financial relationships that could be construed as a potential conflict of interest.
